# Effects of high stocking density on the growth performance, intestinal health and bile salts composition of broiler chickens

**DOI:** 10.3389/fmicb.2025.1542059

**Published:** 2025-02-25

**Authors:** Yuanyang Dong, Yuqi Zheng, Haoyu Liu, Yaru Wang, Jiaqing Cui, Yuan Wu, Lei Yan, Zhiqiang Miao, Miaomiao Han, Chenxuan Huang, Peifeng Li, Yuan Su, Yiru Shen, Junzhen Zhang, Jianmin Yuan, Bingkun Zhang, Jianhui Li

**Affiliations:** ^1^Shanxi Agricultural University College of Animal Science, Taigu, China; ^2^Shandong New Hope Liuhe Group Co., Ltd., Qingdao, China; ^3^Chinese Academy of Agricultural Sciences Poultry Institute, Yangzhou, Jiangsu, China; ^4^China Agricultural University College of Animal Science and Technology, Beijing, China

**Keywords:** high stocking density, broiler, intestinal barrier, intestinal microbiota, bile salt hydrolase

## Abstract

**Introduction:**

The intestinal dysfunction plays an important role in the decreased growth performance of broiler chickens under high stocking density. Gut microbiota plays an important role in maintaining intestinal health. However, the modulation pathway of gut microbiota by regulating the intestinal barrier and histomorphology remains unknown.

**Methods:**

One hundred and forty-four male Arbor Acres broilers (22-d-old) with similar weight were randomly assigned to two treatments: a high (HSD, 20 broilers/m^2^) or low stocking density treatment (LSD, 14 broilers/m^2^), with six replicates per treatment. The experimental period was 20 days, from 22 to 42 days of age.

**Results:**

The final body weight at 42 days of age was lower in the HSD group (*P* = 0.0013) and average daily feed intake (*P* = 0.016) and weight gain of broilers from 22 to 42 days decreased (*P* = 0.012). In the HSD group on day 42, villus height and the ratio of villus height to the crypt depth in the ileum decreased (*P* < 0.05); mRNA expression of tight junction proteins, occludin (*P* < 0.01) and ZO1 (*P* < 0.05), were downregulated; whereas IL-6, TNFα, and NFκB p65 (*P* < 0.05) and IL-1β (*P* < 0.01) were upregulated. The HSD treatment increased the relative abundance of *Lactobacillus* (*P* = 0.045) and decreased that of *Alistipes* (*P* = 0.031). Cecal concentrations of acetic (*P* < 0.05) and butyric acids (*P* < 0.05) decreased. Gut metabolites co-metabolized by the host and gut microbiota were altered in the HSD group, with decreases in glycerophospholipid and tryptophan metabolites negatively correlated with *Lactobacillus* (*P* < 0.05). The metabolite content of conjugated bile acids decreased and free bile acids increased (*P* < 0.05) with HSD. Bile salt hydrolase (BSH) was increased in the intestine of HSD-treated broilers (*P* < 0.01). The total cholic acid content of the HSD group was lower in the jejunum and ileum (*P* < 0.05) but higher in the cecum than in the LSD group (*P* < 0.01).

**Conclusion:**

HSD caused dysbiosis of the intestinal microbiota such as increased *Lactobacillus*, along with enhanced BSH activity and excessive unabsorbed free bile acids. This resulted in ileal epithelial cells damage, inflammation, decreased growth performance of broilers under high stocking densities.

## 1 Introduction

In broiler production, high stocking density (HSD) is a practical strategy for obtaining higher production profit yields per square meter. However, this practice adversely affects growth performance and decreases the daily weight gain, feed intake, and health of broilers (Goo et al., 2019; Magnuson et al., 2020; Wang et al., 2022). Several mechanisms are involved in the HSD-related growth deterioration in broilers. For example, HSD can disrupt the intestinal epithelial barrier, leading to microbiota dysbiosis and dysfunction (Goo et al., 2019; Guardia et al., 2011). The higher percentage and score of necrotic enteritis lesions observed in broilers reared under HSD may have resulted from humoral immune system dysfunction and impairment of commensal bacteria in the intestinal microbiota (Tsiouris et al., 2015).

The chicken cecum harbors a complex microbiome, which is involved in modulating the development and function of the digestive and immune systems (Pan and Yu, 2014). Diverse intestinal microorganisms provide the host with a vast array of enzymes and substrates, which, together with the metabolic capabilities of the host, yield an extensive metabolome for nutrient and energy purposes (Stanley et al., 2013). Several types of metabolites (e.g., short-chain fatty acids, polyamines, bile acids, gases, choline metabolites, tryptophan and indole derivatives, vitamins, and lipids) produced by gut microbiota act in distinct ways to influence various host processes, including intestinal barrier function, gut motility, nutrition absorption, and metabolism (Liu et al., 2022; Rooks and Garrett, 2016). A dysregulated microbiota produces metabolites that translocate from the gut, cross the disrupted gut barrier, and affect various metabolic organs, such as the liver and adipose tissue, leading to local or systemic metabolic inflammation (Tilg et al., 2020).

Studies on microbiota–host metabolic interactions are essential for understanding the roles of intestinal microbes in maintaining host health or in disease development. Specifically, alterations in the intestinal microflora and associated metabolic disorders caused by HSD-induced stress in broilers have not been extensively evaluated. In this study, we investigated the key microbes and metabolites that affect the growth performance and gut health of broilers under HSD-induced stress.

## 2 Materials and methods

### 2.1 Animal ethics

Experimental procedures were conducted in accordance with the guidelines of the Animal Care and Use Committee of Shanxi Agricultural University and approved by the Animal Ethics Committee of Shanxi Agricultural University (approval number: SXAU-EAW-2022P0507001).

### 2.2 Animals and dietary treatment

A total of one hundred forty-four male Arbor Acres broilers (22 days old) were weighed and randomly assigned to the HSD or low stocking density (LSD) treatments, with six replicates (cages) per treatment. The cage area of 1.0 m × 0.7 m was designed for 20 broilers/m^2^ in the HSD group and 14 broilers/m^2^ in the LSD group. Broilers were provided *ad libitum* access to pelleted feed and water. Basal diets were formulated according to Arbor Acres broiler nutritional standards. Table 1 lists the ingredients and nutrient composition of the basal diets for the growth phase (days 22–42). The crude protein content of the feed samples was measured using the Kjeldahl nitrogen determination according to the Chinese National Standard (GB/T 6432-2018). The values of metabolizable energy, available phosphorus, and essential amino acids, including lysine, methionine, cysteine, and threonine, were calculated by referring to the values of each feed material provided in the China Feed Database (2020). The room temperature was maintained at 35°C during the first 3 days, followed by a reduction to 28–30°C during the next 2 week and 25°C for the remainder of the trial. A standard lighting regime was followed: 23 h of light and 1 h of darkness for the first 5 days followed by 20 h of light and 4 h of darkness from day 6 until the end of the trail.

**TABLE 1:** Ingredient and nutrient composition of the basal diets (% air dry).

Ingredients, %	Day 22–42
Corn	61.20
Soybean meal	25.20
Corn gluten meal	3.00
Flour	2.00
Soybean oil	4.84
Dicalcium phosphate	1.20
Limestone	1.05
NaCl	0.30
L-Lysine (78%)	0.36
DL-Methionine (99%)	0.10
Choline chloride (50%)	0.20
Threonine (99%)	0.10
Arginine (98.5%)	0.06
Mineral premix^1^	0.20
Vitamin premix^2^	0.03
Phytase	0.01
Zeolite	0.15
**Nutrient composition, %**	
ME (Kcal/kg)	3,190
CP, %	18.50
Ca, %	0.73
Available phosphorus	0.31
Lysine, %	1.05
Methionine, %	0.40
Methionine+Cysteine, %	0.80
Threonine, %	0.77

^1^The mineral premix provided per kg diet: Cu 8 mg; Zn 75 mg; Fe 80 mg; Mn 100 mg; Se 0.15 mg; I 0.35 mg.

^2^The vitamin premix provided per kg diet: Vitamin A 15000 IU; Vitamin D_3_ 3600 IU; Vitamin K_3_ 3 mg; Vitamin B_1_ 2.4 mg; Vitamin B_2_ 9.6 mg; Vitamin B_6_ 3.6 mg; Vitamin B_12_ 0.03 mg; Vitamin E 30 IU; biotin 0.15 mg; Folic acid 1.5 mg; Pantothenic acid 13.8 mg; Niacin 45 mg.

### 2.3 Growth performance and sample collection

The broilers were weighed by cage (replicate) on D22 and D42. Feed intake, body weight gain, and feed conversion ratio were calculated from day 22 to 42. On D42, one broiler from each cage replicate was randomly selected, Blood was collected from the wing vein and centrifuged at 3,000 × *g* for 15 min at 4°C, and the resultant serum was stored at –20°C until analysis. Then the broilers were administered sodium pentobarbital (30 mg/kg body weight) intracardially, and then killed by jugular exsanguination. Samples of the jejunum and ileum were collected midway, washed with 0.9% physiological saline, and then snap frozen in liquid nitrogen and stored at –80°C. Additional samples of the jejunum and ileum (1 cm) were collected proximal to the ileocecal junction and fixed in 4% (m/v) paraformaldehyde solution for histological examination. The cecal contents were collected aseptically, snap frozen, and stored at –80°C for 16S rRNA sequencing analysis.

### 2.4 Intestinal histomorphological assay

Formalin-fixed jejunum and ileum samples were prepared using the paraffin embedding method. Consecutive tissue sections (5 μm) were stained using hematoxylin and eosin for histomorphological observation. The intestinal villus height (from the tip of the villus to the villus–crypt junction) and crypt depth (from the base of the crypt to the villus–crypt junction) were measured from 10 randomly selected villi and related crypts from one section per chicken, at 40× magnification. The ratio of the villus height to crypt depth (V:C) was then calculated.

### 2.5 Quantitative reverse transcription polymerase chain reaction

Total RNA was extracted from the jejunum and ileum using TRIzol reagent (Takara Biomedical Technology, Beijing, China) and then reversed transcribed into cDNA using a PrimeScript RT Reagent Kit with gDNA Eraser (Perfect Real Time; Takara Biomedical Technology, Beijing, China). Subsequently, the expression levels of the target genes were determined via the quantitative reverse transcription polymerase chain reaction, using the SYBR Premix Ex Taq kit (Tli RNaseH Plus; Takara Biomedical Technology, Beijing, China) according to the manufacturer’s protocols. The primers of the target genes and house-keeping gene are shown in Table 2. The relative gene expression levels were calculated using the 2^–ΔΔCt^ method (Schmittgen and Livak, 2008).

**TABLE 2:** Primer used for real-time PCR.

Gene	Forward primer	Reverse primer	Accession number
β-actin	GCTACAGCTTCACCACCACA	TCTCCTGCTCGAAATCCAGT	NM_205518.1
JAM 1	TGTTCGGAGTCGGAGGGTTCG	AGAGTGTAGGAGGAGTTGCGGAAG	NM_001083366
Occludin	CTGCTCTGCCTCATCTGCTTCTTC	CCATCCGCCACGTTCTTCACC	NM_001013611.2
ZO1	CTTCAGGTGTTTCTCTTCCTCCTC	CTGTGGTTTCATGGCTGGATC	NM_001301025.3
Claudin1	TCCGCAGCAGTTTGGTCA	TCCGCAGCAGTTTGGTCA	NM_001013611.2
Claudin2	CTGCTCACCCTCATTGGA	AACTCACTCTTGGGCTTCTG	NM_001277622.1
IL-1β	CAGAAGAAGCCTCGCCTGGATTC	GCCTCCGCAGCAGTTTGGTC	NM_204524.2
IL-6	GAGGTTGGGCTGGAGGAGGAG	TCTCGCACACGGTGAACTTCTTG	NM_204628.2
IL-22	CTTCTGCTGTTGTTGCTGTTTCCC	GCCAAGGTGTAGGTGCGATTCC	NM_001199614.1
TNFα	CCCAGTTCAGATGAGTTGCCCTTC	GCCACCACACGACAGCCAAG	XM_046927265.1
NfκB P65	ACCACCACCACCACAACACAATG	CAACTCAGCGGCGTCGATGG	NM_001396038.1

JAM1, junctional adhesion molecule 1; ZO1, tight junction protein 1.

### 2.6 Analysis of serum biochemical indexes

The serum levels of total triglyceride, total cholesterol, high-density lipoprotein (HDL), and low-density lipoprotein (LDL) were measured using commercial kits (Nanjing Jiancheng Bioengineering Institute, Nanjing, China). In brief, the total triglyceride level was determined with the glycerol-3-phosphate oxidase–phenol aminophenazone method. The total cholesterol level was determined using the cholesterol oxidase–phenol aminophenazone method. The HDL and LDL levels were determined using the cholesteryl esterase–cholesterol oxidase method with different auxiliary reagents.

### 2.7 16S rDNA amplicon analysis

The cecal contents were collected in the sterile tube snap frozen, and stored at −80°C for 16S rDNA sequencing. Total DNA was extracted using the Microbial Communities kit (Omega Bio TEK, Norcross, GA, USA), and the V3–V4 variable region of 16S rRNA was PCR-amplified using the EZNA^®^ DNA/RNA Isolation Kit according to the manufacturer’s instructions.

The pilot sequencing was performed on the Illumina MiSeq PE300 platform (San Diego, CA, USA). *Fastp* (version 0.20.0)^1^ software was used for quality control of the raw sequences, and *flash* (version 1.2.7)^2^ software was used for splicing with UPARSE software (version 7.1).^3^ The sequences were clustered into operational taxonomic units (OUT) based on 97% similarity and chimeras were excluded. The Silva 16S rRNA database (v138) was aligned with an alignment threshold of 70% using the RDP classifier (version 2.2)^4^ for the taxonomic annotation of each sequence. The abundance information of the OTU was normalized using the sequence number criterion with the smallest sample correspondence. Subsequent α- and β-diversity analyses were performed using normalized data.

### 2.8 Determination of short chain fatty acids concentrations in cecum chyme

Concentrations of short chain fatty acids including acetate, isobutyric acid, butyric acid, isovaleric acid and valeric acid in the cecal chyme were measured using gas chromatography GCMS-7890b-7000d Ultra instrument (Agilent Technologies, Santa Clara, CA, USA). Briefly, cecal chyme (0.5–1 g) was added into a 10 mL centrifuge tube and thawed on ice. An amount of 200 μl 2-ethylbutyric acid solution (1.0 mg/mL) and 5 mL mixed solution of 1% hydrochloric acid and 5% formic acid were added and mixed. The samples were placed in an ice water bath for 30 min with intermittent shaking, then centrifuged at 1,500 rpm for 10 min. The supernatant was transferred to a 1.5 ml centrifuge tube, and then centrifuged at 14,000 rpm for 10 min. The supernatant was filtered with a 0.45 μm pore membrane, and the sample was measured and calculated by the gas chromatography using standard curve method. The data presented in the study are deposited in the NCBI SRA repository, accession number PRJNA868544.

### 2.9 Metabolome analysis of cecal digesta

Cecal digesta was preprocessed using high-throughput tissue crusher Wonbio-96c (Shanghai Wanbo Biotechnology Co., Ltd., Shanghai, China) before the analysis of liquid chromatography–tandem mass spectrometry. Raw liquid chromatography–mass spectrometry data were processed using ProGenesis Qi software (Waters Corporation, Milford, MA, USA) for baseline filtering, peak identification, integration, retention time correction, and peak alignment.

### 2.10 Total bile acid and bile salt hydrolase

The chyme (0.2–1 g) from duodenum, jejunum, ileum, and cecum was homogenized with pre-cold 0.9% saline and centrifuged at 3,000 × *g* for 10 min at 4°C. The supernatant was stored at −20°C for the measurement of total bile acids and bile salt lyase. In brief, total bile acid was determined by enzymatic cycling method in the presence of NADH and a chromophore using commercial kits (Nanjing Jiancheng Bioengineering Institute, Nanjing, China). Bile salt hydrolase (BSH) was determined by ELISA kit (Shanghai Enzyme-linked Biotechnology Co., Ltd., Shanghai, China).

### 2.11 Statistical analysis

The data analysis was conducted using SPSS 26.0 software. Effects of high stocking density on growth performance, intestinal function, and serum lipid-related biochemical parameters, BSH activity and total cholic acid of intestinal digesta were determined using unpaired Student’s *t*-test. Significance was accepted at *P* < 0.05. Cecum microbial phyla, and genera were compared using the Wilcoxon rank-sum test. Differentially expressed metabolites were screened based on a fold change (FC) > 2 or < 0.5 and variable importance in projection > 1.2. The correlations among growth performance, intestinal digesta metabolites, intestinal inflammation-related factors, intestinal tight junction proteins and gut microbiota were analyzed using Spearman’s rank correlation analysis and visualized using Cytoscape 3.10.2.

## 3 Results

### 3.1 Effects of high stocking density on growth performance, intestinal function, and serum lipid-related biochemical parameters

The final body weight (*P* = 0.0013), average daily feed intake (*P* = 0.016), and body weight gain (*P* = 0.012) were lower in the HSD group than in the LSD group; however, no significant difference in the feed conversion ratio (*P* = 0.170) was observed between the two groups (Table 3). The effects of the treatments on the morphological characteristics of the ileum are shown in Table 4; villus height and V:C ratio were substantially lower in the HSD group.

**TABLE 3:** Effect of stocking density on growth performance of broilers from day 22 to 42.

Item^1^	LSD	HSD	SEM	*P*-value
Final body weight on day 42, kg	2.26	2.08	0.04	0.013
Average daily feed intake, g/d per bird	118	110	2	0.016
Body weight gain, g/d per bird	81	73	2	0.012
Feed conversion ratio, g/g	1.45	1.51	0.02	0.17

^1^LSD, low stocking density; HSD, high stocking density.

**TABLE 4:** Effect of stocking density on ileal morphology of broilers.

Item^1^	LSD	HSD	SEM	*P*-value
Villus height, μm	791.8	492.9	7.4	< 0.01
Crypt depth, μm	96.1	102.8	2.1	0.474
Ratio of villus height to crypt depth	8.37	4.91	0.13	< 0.01

^1^LSD, low stocking density; HSD, high stocking density.

**FIGURE 1:**
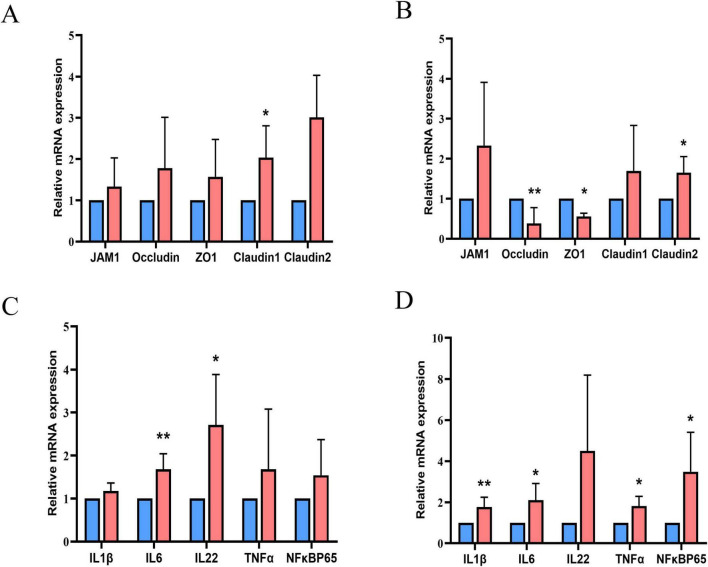
Relative mRNA expression levels of genes related to intestinal barrier function in the jejunum **(A)** and ileum **(B)**, and genes related to inflammation in the jejunum **(C)** and ileum **(D)**. Blue columns for the group LSD; red columns for the group HSD.

Figures 1A–D shows the relative mRNA expression levels of genes related to intestinal barrier function and inflammation in the jejunum and ileum. HSD upregulated the mRNA expression levels of claudin-1 in the jejunum and claudin-2 in the ileum, but downregulated those of occludin and ZO-1 in the ileum (*P* < 0.01). The mRNA expression levels of interleukin (IL)-6 and IL-22 in the jejunum were substantially higher in the HSD than in the LSD group. The expression of IL-1β, IL-6, tumor necrosis factor-alpha (TNFα), and nuclear factor-kappa B (NFκB) p65 mRNA in the ileum was higher in the HSD group (*P* < 0.01). HSD treatment increased serum LDL levels (*P* = 0.013) and there was a tendency toward higher serum content of total triglycerides (TG) (*P* = 0.05) than in the LSD group (Table 5). However, serum total cholesterol (TC) and HDL levels were not influenced by HSD (*P* > 0.05).

**TABLE 5:** Effect of stocking density on serum lipid-related biochemical parameters of broilers.

Item^1^	LSD	HSD	SEM	*P*-value
Total cholesterol (mmol/L)	2.86	3.2	0.113	0.137
Total triglycerides (mmol/L)	0.38	0.44	0.017	0.05
Low-density lipoprotein (mmol/L)	0.53	0.76	0.049	0.013
High-density lipoprotein (mmol/L)	2.39	2.35	0.056	0.76

^1^LSD, low stocking density; HSD, high stocking density.

### 3.2 Cecal microbiota

**FIGURE 2:**
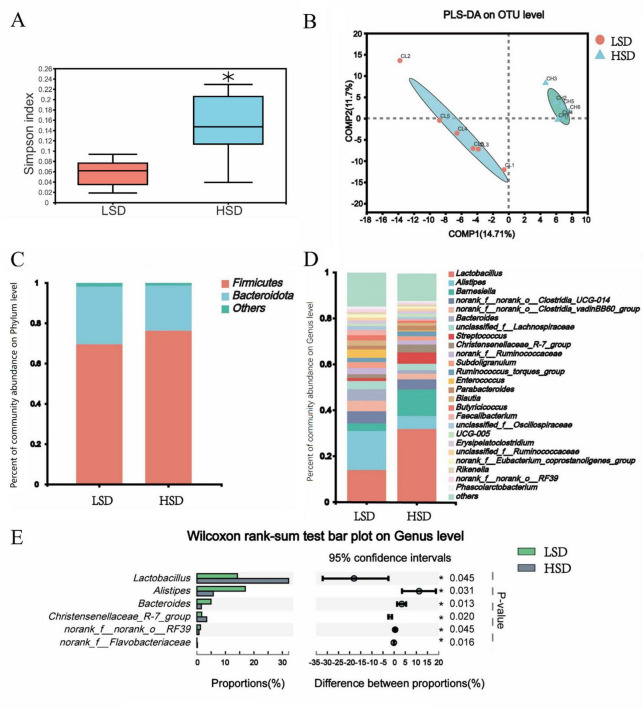
**(A)** Elevated Simpson index was observed in the HSD group; **(B)** High stocking density caused significant changes of cecal microbiota β-diversity; microbial composition difference at phylum **(C)** and genus level **(D)**; **(E)** The different cecal microbiota based on Wilcoxon rank-sum test.

The Simpson index was markedly elevated in the HSD group (Figure 2A) and the partial least-squares discriminant analysis of β-diversity indicated substantial differences in microbial communities between the two treatment groups (Figure 2B). At the phylum level, the relative abundance of Firmicutes was higher in the HSD group and fewer Bacteroidetes were observed (Figure 2C). Among the top 25 genera studied, *Lactobacillus* was present at a higher abundance (*P* = 0.045) in the HSD group, whereas *Alistipes* was present at a lower abundance than in the LSD group (Figures 2D, E). The cecal concentrations of acetic and butyric acids were substantially reduced under HSD, whereas concentrations of isobutyric, isovaleric, and valeric were similar at both stocking densities (Table 6).

**TABLE 6:** Effect of stocking density on the content of short chain fatty acids in cecal digesta.

Item^1^	LSD	HSD	SEM	*P*-value
Acetic acid, μg/mg	3.54	2.14	0.52	0.023
Propionic acid, μg/mg	0.82	0.69	0.15	0.436
Isobutyric acid, μg/mg	0.14	0.15	0.02	0.711
Butyric acid, μg/mg	1.48	0.86	0.26	0.045
Isovaleric acid, μg/mg	0.17	0.16	0.03	0.762
Valeric acid, μg/mg	0.16	0.14	0.02	0.460

^1^LSD, low stocking density; HSD, high stocking density.

### 3.3 Cecal digesta metabolites and their correlation with the microbiota

**FIGURE 3:**
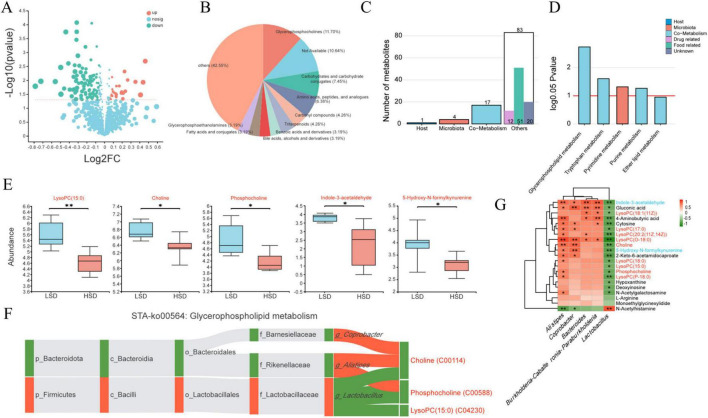
**(A)** Overall regulation situation for cecal digesta metabolites identified between the HSD and LSD groups; **(B)** top 10 categories for the differential metabolites based on the Human Metabolome Database; **(C)** the number of metabolites generated by host, microbiota or both using metabolite tracing analysis; **(D)** related metabolic pathway for the metabolites generated by host, microbiota or both; **(E)** differential co-metabolized metabolites caused by HSD treatment; the correlation between differential microbiota and differential metabolites using heatmap **(G)** and metabolites in glycerophospholipid metabolism **(F)**.

In total, 121 differential metabolites were identified between the HSD and LSD groups, with 27 increased and 94 decreased following the HSD treatment (Figure 3A). The differential metabolites were classified using the Human Metabolome Database (Figure 3B). Among the top 10 categories, glycerophosphocholines accounted for the largest proportion (11.70%), followed by the metabolites of carbohydrates, amino acids, carbonyl compounds, triterpenoids, benzoic acid, bile acids, fatty acids, and glycerophosphoethanolamines. Metabolite tracing analysis against the Kyoto Encyclopedia of Genes and Genomes (KEGG) and Human Metabolome databases revealed four differential metabolites that were metabolized by microbes only, including tryptophan, cytosine, and *N*-[(3a,5b,7b)-7-hydroxy-24-oxo-3-(sulfooxy)cholan-24-yl]-glycineṠeventeen metabolites were co-metabolized by microbes and hosts [including lysophospholipid choline-like metabolites, phosphocholine, choline, D-pantothenic acid, 4-aminobutyric acid, and tryptophan metabolites (5-hydroxy-*N*-formylkynurenine and indole-3-acetaldehyde)] (Figure 3C). According to KEGG pathway enrichment analysis, microbially-metabolized metabolites were enriched in the pyrimidine metabolism pathway, whereas metabolites co-metabolized by microbes and the host were enriched in glycophospholipid, tryptophan, and purine metabolism pathways (Figure 3D).

Co-metabolized lysoPC(15:0) (a lysophospholipid choline-like metabolite), choline, phosphocholine, indole-3-acetaldehyde, and 5-hydroxy-*N*-formylkynurenine levels were substantially reduced by HSD (Figure 3E). Choline, phosphocholine, and lysoPC(15:0) metabolites were strongly negatively correlated with *Lactobacillus*, whereas choline and phosphocholine were positively correlated with *Alistipes* (Figure 3F). Most of the differential metabolites (including phosphocholine, choline, 4-aminobutyric acid, tryptophan metabolites, 5-hydroxy-*N*-formylkynurenine, indole-3-acetaldehyde, and seven lysophospholipid choline metabolites) were negatively correlated with *Lactobacillus*, except for *N*-acetylhistamine (Figure 3G). In contrast, most metabolites, including choline, phosphocholine, and six lysophospholipid choline metabolites were positively correlated with *Alistipes*.

### 3.4 Relationship between bile acid metabolism and intestinal microorganisms

**FIGURE 4:**
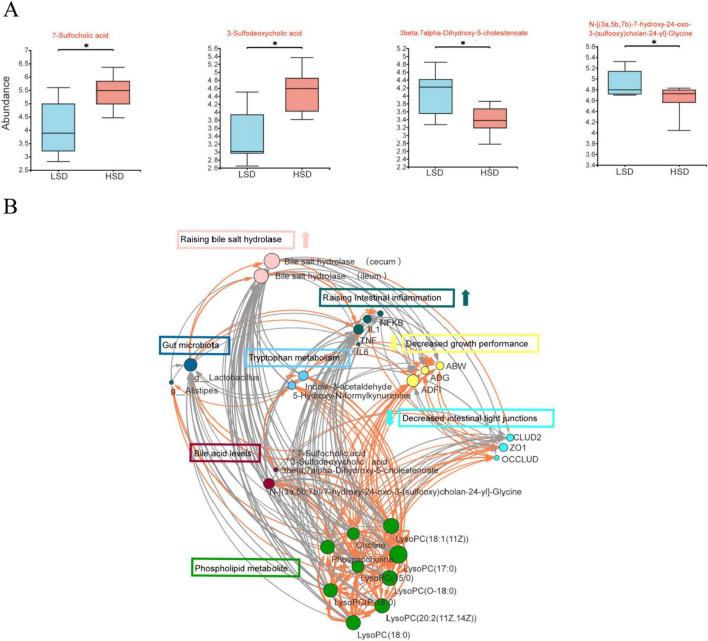
**(A)** intestinal bile acid metabolites among the differential cecal metabolites was examined between LSD and HSD groups; **(B)** the correlations between growth performance, intestinal barrier, inflammation status, cecal microorganisms, and bile acid metabolites using network analysis.

Of the differential cecal metabolites, intestinal bile acid metabolites between the two treatment groups were examined further. The contents of two sulfated cholic acids (7-sulfocholic and 3-sulfodeoxycholic acids) were substantially higher, whereas those of a secondary bile acid glycine conjugate {*N*-[(3a,5b,7b)-7-hydroxy-24-oxo-3-(sulfooxy) cholan-24-yl]-glycine} and goose deoxycholic acid precursor (3β,7α-dihydroxy-5-cholestenoate) were substantially lower in the HSD group (Figure 4A).

HSD treatment increased the levels of BSH in the duodenum, jejunum, ileum, and cecum (*P* < 0.01) (Table 7). Additionally, the HSD decreased the total cholic acid content in the jejunum and ileum (*P* < 0.05), but increased the amount of total cholic acid in the cecum compared with the LSD group (*P* < 0.01) (Table 8).

**TABLE 7:** Effect of stocking density on the content of bile salt hydrolase in each intestinal segment.

Item^1^		LSD	HSD	SEM	*P*-value
BSH, mmol/g	Duodenum	66.3	77.8	2.4	0.002
	Jejunum	40.6	54.4	4.0	0.006
	Ileum	45.6	65.0	2.7	< 0.001
	Cecum	38.0	53.1	4.0	0.004

^1^LSD, low stocking density; HSD, high stocking density.

**TABLE 8:** Effect of stocking density on the content of total bile acid in each intestinal segment.

Item^1^		LSD	HSD	SEM	*P*-value
Total bile acid, mmol/g	Duodenum	162.7	148.9	19.9	0.51
	Jejunum	160.0	52.8	19.0	0.001
	Ileum	58.0	17.5	7.3	< 0.001
	Cecum	104.0	159.2	21.4	0.027

^1^LSD, low stocking density; HSD, high stocking density.

To elucidate the correlations between growth performance, intestinal barrier, inflammation status, cecal microorganisms, and bile acid metabolites, network analysis was performed using Cytoscape software Version 3.10.2. Growth performance was positively correlated with phospholipid metabolites; tryptophan metabolism; intestinal tight junction proteins, ZO1, and occludin; were negatively correlated with intestinal inflammation and BSH (Figure 4B). As the major differential microorganism between the two treatment groups, *Lactobacillus* was positively correlated with BSH and negatively correlated with phospholipid metabolites. BSH was negatively correlated with growth performance; phospholipid metabolites; tryptophan metabolism; intestinal tight junction proteins, ZO1 and occludin; and positively correlated with TNFα.

## 4 Discussion

In this study, we explored the mechanisms underlying HSD-induced stress in broilers. As expected, the HSD treatment decreased the average body weight gain and final body weight of the animals (Goo et al., 2019; Magnuson et al., 2020; Li et al., 2019).

In broilers, gastrointestinal tract development is an important aspect of growth and is intricately related to nutrient utilization (Choct, 2009). Intestinal morphological changes such as a shorter villus height result in a decreased surface area for nutrient absorption; a reduced V:C ratio causes a higher demand for energy and protein for gut maintenance (Choct, 2009). The villus height and V:C ratio in the current study decreased under HSD, indicating that the ability of the small intestine to absorb nutrients was compromised and the demand for energy would therefore be concomitantly higher.

The intestines facilitate the absorption of nutrients and function as a barrier against potential pathogens. As important components of the intestinal barrier, tight junctions are composed of transmembrane and cytosolic proteins, including occludin, junctional adhesion molecules (decreased paracellular permeability), claudin-1 and claudin-2 (forming charge-selective paracellular pores in leaky epithelial tissue), and ZOs (interacting with transmembrane proteins and the cytoskeleton as linker proteins) (Chelakkot et al., 2018; Ulluwishewa et al., 2011). In the present study, HSD decreased the mRNA expression of occludin and ZO-1, suggesting an increase in intestinal permeability and susceptibility to pathogenic bacteria. An increase in mRNA expression of claudin-1 and -2 was detected in HSD broilers. In a mouse model of intestinal claudin-1 overexpression, the animals were susceptible to colonic inflammation and showed impaired recovery following dextran sulfate sodium-induced colitis (Pope et al., 2014). In a mouse model of *Citrobacter rodentium* infection, IL-22 induced the upregulation of claudin-2, which caused diarrhea (Tsai et al., 2017). In the present study, HSD induced an increase in the mRNA expression of inflammation-related cytokines (IL-1β, IL-6, TNFα, and NFκB p65) in the jejunum and ileum. Thus, disturbed tight junctions and inflammation may contribute to the stress status and morphological changes caused by HSD in the small intestine.

In modern poultry production, dietary oil supplementation is commonly used to increase the dietary energy levels (Poorghasemi et al., 2013). The triacylglycerols from the diet and storage in the liver depend on the availability of plasma lipids (Alvarenga et al., 2011). In the current study, HSD increased the LDL and tended to increase the serum TG levels. The increased serum LDL and TG levels induced by stocking density may be related to the deposition of abdominal fat, which could lead to impaired liver function (Xie et al., 2023). HSD treatment increased LDL levels without influencing the total cholesterol or HDL content. Since total cholesterol comprises VLDL, LDL, and HDL, the HSD-induced decrease in serum VLDL indicates limited utilization of crude fat.

Although gut bacteria can drive immune activation, intestinal inflammation shapes the gut microbiota and contributes to dysbiosis (Ni et al., 2017). In the current study, diversity analysis revealed a difference in the microbiota of the two treatment groups, particularly a decrease in various anaerobes (*Alistipes* and *Bacteroides*) and an increase in facultative anaerobes (*Lactobacillus*) in the cecum of HSD broilers. Many anaerobic intestinal microorganisms, such as *Bacteroides* and *Alistipes*, can produce short-chain fatty acids (e.g., acetic, propionic, and butyric acids) by fermenting dietary fiber (Adil and Magray, 2012). The reduced abundance of anaerobes (*Bacteroides* and *Alistipes*) in HSD broilers may have contributed to the decreased acetic and butyric acid content in the intestinal tract. Depletion of the butyric acid-producing microbiota results in luminal oxygenation (Kelly and Colgan, 2016). Thus, in the present study, the high-O_2_ condition in the gut lumen caused by the reduced abundance of butyric acid-producing microorganisms may have led to an increased abundance of *Lactobacillus* under HSD conditions.

Metabolites co-metabolized by the host and gut microbiota (such as glycerophospholipid and tryptophan metabolism) were considerably altered in the HSD group. As essential components of cell membranes, glycerophospholipids are ubiquitous in all tissues and participate in various metabolic processes (Castro-Gómez et al., 2015). Nine glycerophospholipid metabolites [including lysoPC(15:0), choline, and phosphocholine] were substantially decreased and were negatively correlated with *Lactobacillus* in the HSD group.

Many microbes (e.g., *Escherichia coli* and *Bacteroides*) residing in the intestine can use tryptophan as a nitrogen source and metabolize it into indole and indole derivatives. These are signaling molecules with anti-inflammatory activity that can restore intestinal flora disturbances (Konopelski and Ufnal, 2018; Sun et al., 2020). In the current study, the contents of tryptophan metabolites (indole-3-acetaldehyde and 5-hydroxy-*N*-formylkynurenine) were substantially decreased in the HSD group, negatively correlated with increased *Lactobacillus* abundance, and positively correlated with decreased *Bacteroides* abundance in the cecum. A reduction in tryptophan metabolites due to tryptophan deficiency is closely related to intestinal imbalance, which may lead to inflammatory bowel disease (Sun et al., 2020).

Considering our findings of disrupted intestinal morphology and intestinal dysbiosis, we focused on the relationships between lipid digestion, intestinal tight junctions, and intestinal microbiota. Fat digestion and absorption are complex processes, and emulsification is central to efficient fat utilization. In poultry, bile salts are conjugated with taurine in the liver, increasing their water solubility and decreasing their cellular toxicity. Conjugated bile acids display stronger fat-emulsifying activity than free bile acids, which cannot be reabsorbed into the ileum and are excreted. In birds, poor fat digestion may be due to the reduced recycling of bile salts and their concomitant low concentrations (Ravindran et al., 2016). In the current study, the content of *N*-[(3a,5b,7b)-7-hydroxy-24-oxo-3-(sulfooxy)cholan-24-yl]-glycine (a metabolite of conjugated bile acids) decreased, whereas that of 7-sulfodeoxycholic and 3-sulfodeoxycholic acids (metabolites of free bile acids) increased in the HSD group.

BSH is a key enzyme that catalyzes the transformation of conjugated bile acids into their free forms (Winston and Theriot, 2020). Unabsorbed bile acids enter the distal intestine and undergo deconjugation through the BSH activity of gut microbes (Winston and Theriot, 2020). Most BSH enzymes are detected in several genera of gastrointestinal, autochthonous micro-organisms in animals, including *Bifidobacterium*, *Lactobacillus*, *Enterococcus*, and *Streptococcus*. Of these bacteria, *Lactobacilli* constitute an important aspect of animal intestinal microbiota and contribute to the majority of the total BSH activity *in vivo* (Dong and Lee, 2018). *Lactobacillus* populations are the major producers of BSH in the small intestine (Begley et al., 2006). HSD-induced a higher *Lactobacillus* abundance and the concomitantly elevated BSH activity in all intestinal segments may regulate the hydrolysis of excessive conjugated bile acids in the distal intestine. In the current study, total cholic acid content was confirmed to decrease in the anterior gut and increase in the distal gut of HSD broilers, which may lead to increased fecal loss of bile acids. Furthermore, 3β,7α-dihydroxy-5-cholestenoate, which is the precursor of the bile acid, chenodeoxycholic acid, which is synthesized via the CYP27A1 pathway (Chiang and Ferrell, 2019), was decreased in the HSD group, indicating that bile acid synthesis via this anabolic pathway was inhibited by HSD. Excess free bile acids in the intestine can have toxic effects on the intestinal epithelial cells, leading to cell membrane damage, disruption of intestinal tight junctions, and inflammation (Pavlidis et al., 2015). These effects are exacerbated by dysbiosis of the gut microbiota. BSH enrichment is positively correlated with colitis (Parasar et al., 2019). Based on these results, the increased BSH content derived from *Lactobacillus* is one of the main reasons for the disrupted intestinal barrier and inflammation caused by high stocking densities. Similarly, the growth-promoting effects of antibiotic growth promoters are strongly correlated with reduced BSH (Dong and Lee, 2018). Antibiotic growth promoters inhibit BSH activity by reducing the abundance of *Lactobacillus* in the intestine, with a concomitant increase in animal weight (Lin, 2014).

## 5 Conclusion

In conclusion, HSD treatment leads to the disruption of intestinal tight junction proteins, such as occludin, claudin, and ZO1, as well as intestinal inflammation in broilers. The enhanced BSH activity derived from dysbiosis of the intestinal microbiota under high stocking density disrupted bile acid metabolism, leading to excessive unabsorbed free bile acids in the distal gut. This resulted in toxic damage to intestinal epithelial cells, intestinal inflammation, and limited fat utilization. Therefore, BSH may be a pivotal modulator of intestinal damage and decreased growth performance of broilers under high stocking densities.

## Data Availability

The data presented in the study are deposited in the NCBI SRA repository, accession number PRJNA868544.

## Footnotes

1
https://github.com/opengene/fastp

2
http://www.cbcb.umd.edu/software/flash

3
http://drive5.com/uparse/

4
http://rdp.cme.msu.edu/
